# PAF1 complex interactions with SETDB1 mediate promoter H3K9 methylation and transcriptional repression of *Hoxa9* and *Meis1* in acute myeloid leukemia

**DOI:** 10.18632/oncotarget.25204

**Published:** 2018-04-24

**Authors:** James Ropa, Nirmalya Saha, Zhiling Chen, Justin Serio, Wei Chen, Dattatreya Mellacheruvu, Lili Zhao, Venkatesha Basrur, Alexey I. Nesvizhskii, Andrew G. Muntean

**Affiliations:** ^1^ Department of Pathology and The University of Michigan Medical School, Ann Arbor, Michigan, USA; ^2^ Department of Computational Medicine and Bioinformatics, University of Michigan Medical School, Ann Arbor, Michigan, USA; ^3^ Department of Biostatistics, University of Michigan School of Public Health, Ann Arbor, Michigan, USA

**Keywords:** polymerase associated factor complex, H3K9 methyltransferase, protein-protein interaction, transcription, leukemia

## Abstract

The Polymerase Associated Factor 1 complex (PAF1c) is an epigenetic co-modifying complex that directly contacts RNA polymerase II (RNAPII) and several epigenetic regulating proteins. Mutations, overexpression and loss of expression of subunits of the PAF1c are observed in various forms of cancer suggesting proper regulation is needed for cellular development. However, the biochemical interactions with the PAF1c that allow dynamic gene regulation are unclear. We and others have shown that the PAF1c makes a direct interaction with MLL fusion proteins, which are potent oncogenic drivers of acute myeloid leukemia (AML). This interaction is critical for the maintenance of *MLL* translocation driven AML by targeting MLL fusion proteins to the target genes *Meis1* and *Hoxa9*. Here, we use a proteomics approach to identify protein-protein interactions with the PAF1c subunit CDC73 that regulate the function of the PAF1c. We identified a novel interaction with a histone H3 lysine 9 (H3K9) methyltransferase protein, SETDB1. This interaction is stabilized with a mutant CDC73 that is incapable of supporting AML cell growth. Importantly, transcription of *Meis1* and *Hoxa9* is reduced and promoter H3K9 trimethylation (H3K9me3) increased by overexpression of SETDB1 or stabilization of the PAF1c-SETDB1 interaction in AML cells. These findings were corroborated in human AML patients where increased *SETDB1* expression was associated with reduced *HOXA9* and *MEIS1*. To our knowledge, this is the first proteomics approach to search for CDC73 protein-protein interactions in AML, and demonstrates that the PAF1c may play a role in H3K9me3-mediated transcriptional repression in AML.

## INTRODUCTION

The PAF1c is a highly conserved complex that was first identified in yeast as a transcriptional regulating complex that co-purified with RNA polymerase II (RNAPII) [1, 2]. The PAF1c is composed of several subunits: PAF1, CDC73, CTR9, LEO1, RTF1, and the mammalian specific subunit WDR61 [3–5]. While lacking any known catalytic activity itself, the PAF1c plays a critical role in the dynamic regulation of epigenetic landscapes at gene loci. The complex modulates epigenetic landscapes via protein-protein interactions with epigenetic modifying proteins [6, 7]. For example, the PAF1c has been shown to be important for histone H3 lysine 4 trimethylation (H3K4me3) and histone H3 lysine 79 dimethylation (H3K79me2) modifications through its interaction with Mixed Lineage Leukemia (MLL) histone methyltransferase and the Super Elongation Complex (SEC) [8–10]. Additionally, the PAF1c is necessary for H2B monubiquitination (H2Bub) through its recruitment of ringer finger proteins RNF20/40 and the ubiquitin ligase RAD6 [11–20]. The PAF1c and these epigenetic modifications that it modulates are critical for transcriptional elongation at a subset of genes in yeast and mammals [8, 13, 16, 21–25].

While much of the work on the PAF1c has demonstrated a role in active transcription elongation, there is also evidence that subunits of the PAF1c are involved in transcriptional repression. For instance, the PAF1 subunit is necessary for proper promoter-proximal pausing of RNAPII [26]. Further, the hyperactivation of a subset of transcriptional enhancers is restrained by PAF1 illustrating a role in transcriptional repression via enhancer regulation [27]. Furthermore, the PAF1c subunit CDC73 can transcriptionally repress oncogenic targets, such as *MYC* and *CCND1* [28, 29]. Additionally, CDC73 has been shown to promote *CCND1* promoter H3K9me3 by recruitment of the H3K9 methyltransferases SUV39H1 or G9a [30]. This may contribute to tumor suppressor activity attributed to CDC73, which is mutated in hyperparathyroidism-jaw tumor syndrome (HPT-JT), exhibiting a loss of function mutation in more than 80% of these malignancies [31, 32]. These studies suggest that the complex may be dynamically regulated to function as a transcriptional co-activator or co-repressor in different cellular contexts.

In contrast to its role in HPT-JT, our work and others revealed a requisite role for the PAF1c complex in leukemias harboring a *MLL (KMT2a)* translocation [9, 10, 33]. MLL is a histone methyltransferase that deposits the H3K4me3 modification associated with promoter regions of actively transcribed genes. *MLL* is involved in chromosomal translocations with a variety of gene fusion partners that result in oncogenic MLL fusion proteins that drive transcription of genes critical for leukemogenesis, such as *MEIS1* and *HOXA9* [34–36]. In a study to understand the regulation of these leukemogenic target genes, we and others found a direct physical interaction between the PAF1c and MLL or MLL-fusion proteins, which are necessary for MLL mediated leukemias [9, 10]. Importantly, disruption of the PAF1c-MLL interaction selectively inhibits the growth of MLL leukemias but is tolerated by normal hematopoietic cells pointing to cancer specific functions for the PAF1c [33]. Despite the importance of the PAF1c in transcriptional regulation of critical leukemic genes, the biochemical regulation of the PAF1c that allows for the dynamic regulation of target genes, such as *Hoxa9* and *Meis1*, remains poorly understood.

Here, we explore the biochemical regulation of the PAF1c in AML through an Affinity Purification-Mass Spectrometry (AP-MS) approach using CDC73 as bait. We identified both known and novel protein-protein interactions with the PAF1c. These interacting partners included a group of H3K9 methyltransferases, including a novel interacting partner SETDB1. H3K9 methyltransferases are epigenetic modifying proteins associated with transcriptional repression and heterochromatin formation [37]. There is also emerging evidence that SETDB1 plays a role in mediating H3K9me3 at dynamically regulated gene loci, such as the ID2 promoter [38–43]. The developmental *HoxA* gene cluster is also regulated by H3K9me3 in embryonic stem cells and melanoma cells [38, 44]. Despite the importance of the *HoxA* gene cluster and co-factor *Meis1* in AML, we do not understand the role of H3K9me3 in regulating these genes in leukemic cells. In this study, we identified SETDB1 as a novel PAF1c interacting protein and explored the role of this interaction in modulating transcription of the known PAF1c pro-leukemic target genes *Hoxa9* and *Meis1*.

## RESULTS

### Point mutations in CDC73 disrupt AML cell growth without affecting PAF1c complex integrity

**Figure 1:**
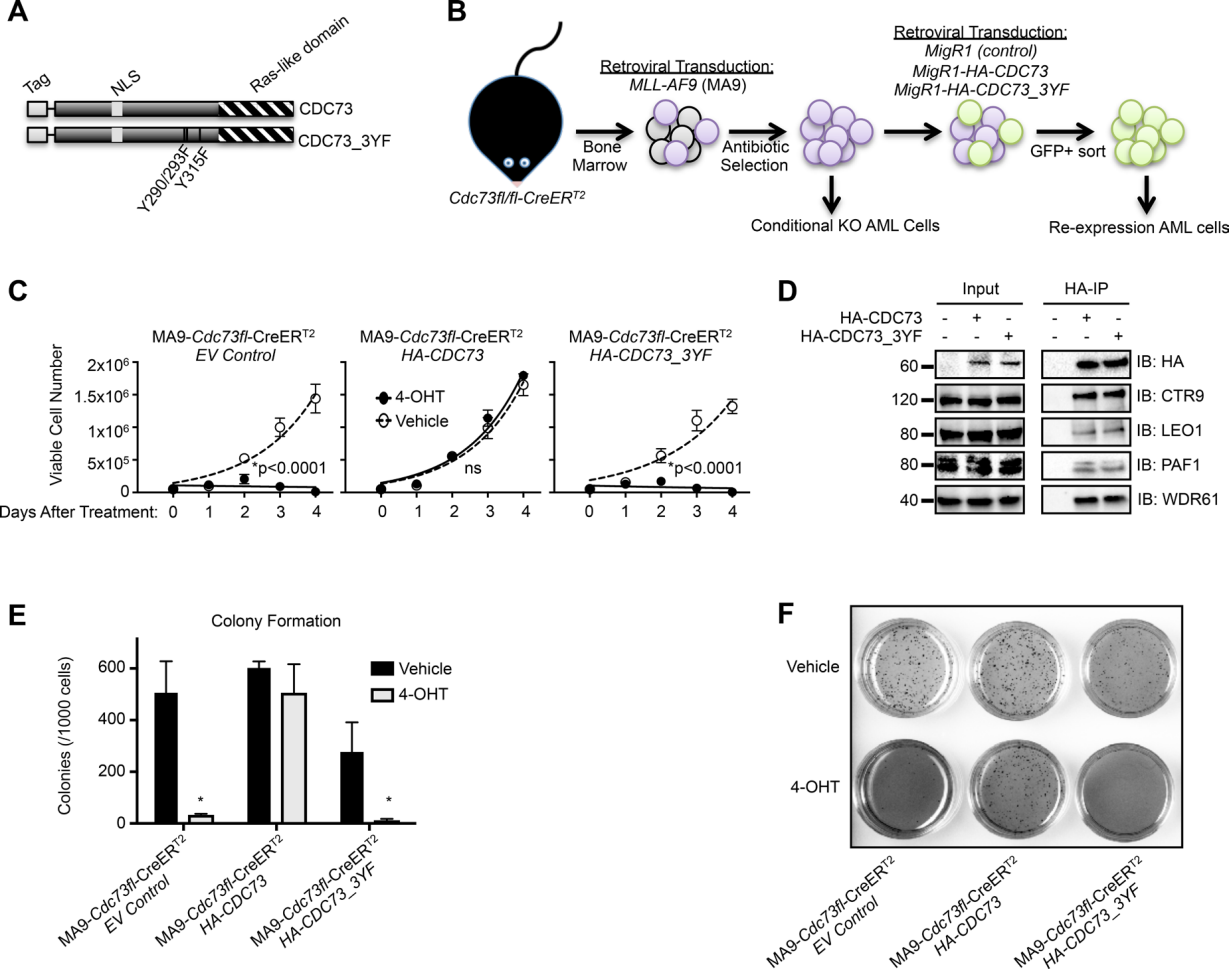
A CDC73 tyrosine mutant does not support AML cell growth **(A)** Schematic of HA-CDC73 showing known domains of CDC73 and the location of the three tyrosine mutations introduced to make CDC73_3YF. **(B)** Diagram representing the workflow for the generation of MLL-AF9 transformed *CDC73fl/fl*-CreERT2(MA9-*Cdc73fl*-CreER^T2^) CDC73 or CDC73_3YF re-expression cells, as well as MigR1 control cells (EV Control). **(C)** The indicated MLL-AF9 transformed *CDC73fl/fl*-CreER^T2^ CDC73 re-expression cells were plated on day 0 in the presence of 4-OHT or vehicle control. Viable cells were counted every day for four days. Shown is one representative assay of n>5 biological replicates, 3 technical replicates each. Statistical analysis performed was 2-way ANOVA with post hoc multiple t-tests. **(D)** Wild type and CDC73_3YF were immunoprecipitated following transient transfection of HEK293T cells. HA-IPs were performed followed by immunoblots with the indicated antibodies for PAF1c components. **(E)** The indicated MLL-AF9 transformed *CDC73fl/fl*-CreER^T2^ CDC73 re-expression cells were pretreated with 4-OHT or vehicle for one day prior to being plated in semisolid methylcellulose. Colonies were counted after 7 days (n=2 biological replicates, 2 technical replicates each). Welch’s two-tailed paired t-test was used to compare 4-OHT treated cells to vehicle treated cells. **(F)** Representative 1x magnification images of INT stained colonies that are quantified in Figure 1E. ^*^p<0.05.

Recent studies have linked the PAF1c subunit Cdc73 with the WNT, Hedgehog and Notch signaling pathways through protein interactions with β-catenin, Gli1 and Notch intracellular domain [45, 46]. A tyrosine mutational analysis has identified a trio of tyrosine residues on CDC73 that regulate its interaction with β-catenin. Mutation of these residues to phenylalanine stabilizes the CDC73 interaction with β-catenin and enhances WNT signaling in gastric carcinoma cells [45, 47]. A critical role for β-catenin in MLL rearranged leukemias prompted us to investigate this triple tyrosine to phenylalanine mutant, CDC73-Y290/293/315F (CDC73_3YF) (Figure 1A) in MLL-AF9 transformed AML cells (Figure 1B) [48–50]. As reported, CDC73_3YF displayed enhanced interaction with β-catenin compared to wild type CDC73 following transient transfection of HEK293T cells and immunoprecipitation (IP) of β-catenin (Supplementary Figure 1A). To explore the biological impact of CDC73_3YF in AML cells, we transduced *Cdc73fl/fl*-CreER^T2^ mouse bone marrow with MLL-AF9 packaged retrovirus to generate stable AML cell lines that can be induced to genetically excise *Cdc73* by treatment with 4-hydroxytamoxifen (4-OHT) (Figure 1B, Supplementary Figure 1C) [51]. 4-OHT treatment results in almost complete loss of the CDC73 protein by 48 hours (Supplementary Figure 1D) [25, 33]. We used retroviral transduction to stably express *CDC73* or *CDC73_3YF* in *Cdc73fl/fl*-CreER^T2^ AML cells to test the rescue capacity of CDC73_3YF upon loss of *Cdc73* (Figure 1B). We confirmed expression of the tagged CDC73 and CDC73_3YF (Supplementary Figure 1B). Upon deletion of *Cdc73*, we confirmed that CDC73 protein was reduced to <5% of vehicle treated cells (Supplementary Figure 1D). Expression of wild type CDC73 or CDC73_3YF following 4-OHT treatment resulted in protein levels at about 50% that of endogenous Cdc73 observed in vehicle treated cells expressing an empty vector control (MigR1) (Supplementary Figure 1D). Proliferation assays demonstrate that, following excision of *Cdc73*, MLL-AF9 cells expressing an empty vector (MigR1) exhibit a significant reduction in cell proliferation, whereas re-expression of CDC73 fully rescued proliferation similar to that of cells treated with vehicle control (Figure 1C). Surprisingly, despite stabilized interaction with β-catenin, CDC73_3YF failed to rescue cellular proliferation similar to MigR1 control cells (Figure 1C). To confirm that CDC73_3YF is capable of binding to the other components of the PAF1c, we transiently transfected HEK293T cells with *HA-CDC73* or *HA-CDC73_3YF* and performed IP-western blots. We observed co-precipitation of CTR9, LEO1, PAF1, and WDR61 with both CDC73 and CDC73_3YF (Figure 1D). We also confirmed PAF1c co-purification with CDC73_3YF in M1 mouse AML cells that stably express retroviral *FLAG-CDC73_3YF* (Supplementary Figure 1E). We further explored the effects of re-expression of CDC73_3YF on the colony forming unit ability of AML cells. Following excision of *Cdc73*, MLL-AF9 cells expressing an empty vector showed a significant reduction in colony forming unit capacity. This phenotype was rescued by re-expression of CDC73 but not CDC73_3YF suggesting this mutant does not support leukemic colony forming unit potential (Figure 1E, 1F). We further observed that HA-CDC73 and HA-CDC73_3YF are present in the chromatin fraction of transiently transfected HEK293T cells (Supplementary Figure 1F). These data indicate that the CDC73_3YF mutant assembles into the PAF1c and stabilizes interaction with β-catenin, but does not support proliferation and colony formation capacity of AML cells.

### CDC73_3YF stabilizes interaction with H3K9 methyltransferases SETDB1 and G9a

**Figure 2:**
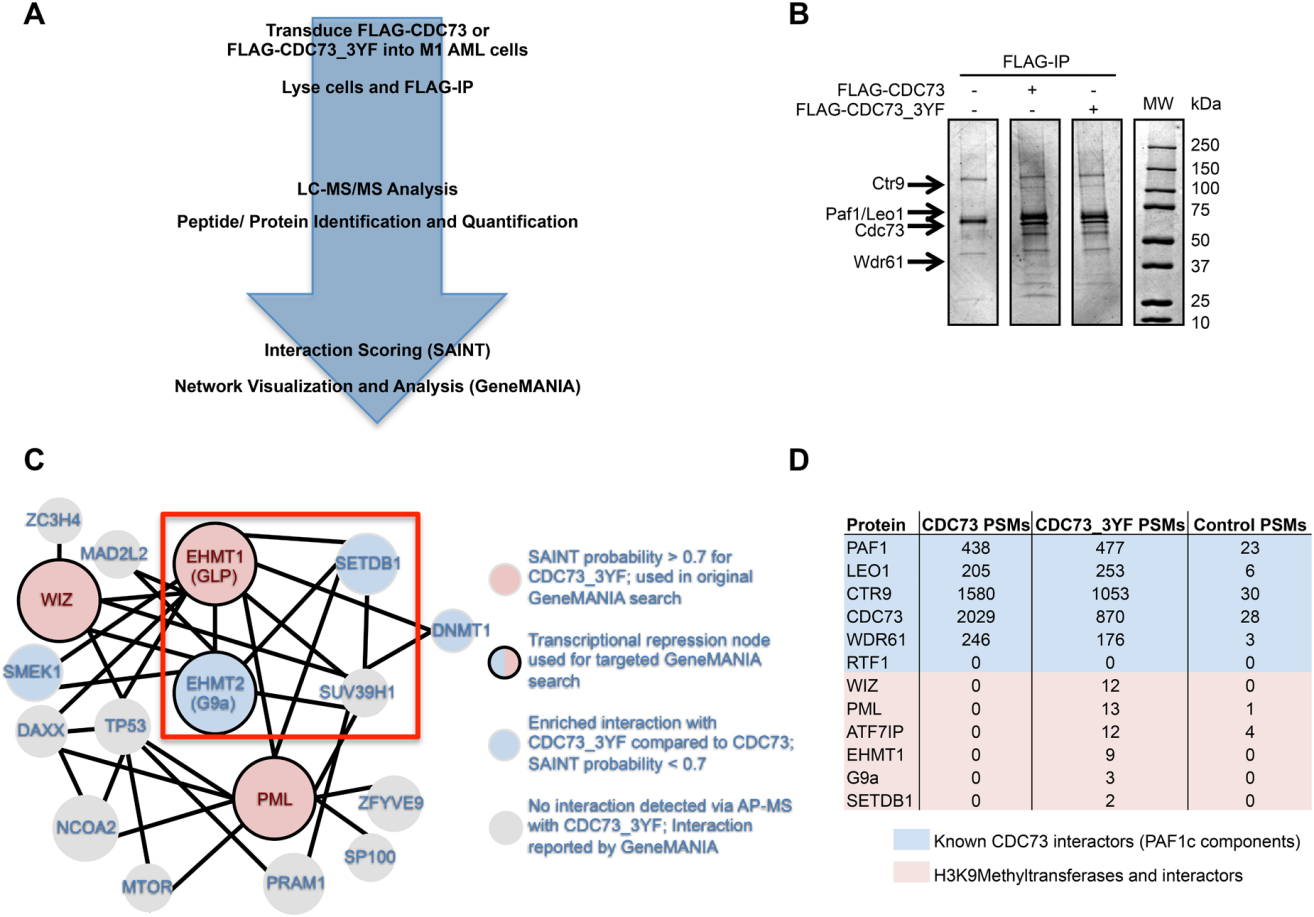
CDC73 and CDC73_3YF have overlapping and distinct interactomes **(A)** Schematic demonstrating the approach for the AP-MS experiment to determine interaction partners of CDC73 and CDC73_3YF in M1 mouse leukemia cells. **(B)** FLAG-IPs of HEK293T cells transiently transfected with CDC73 or CDC73_3YF were eluted and run on a gel that was stained with Coomassie Blue. Arrows indicate the appropriate size for protein bands for the given PAF1c components. **(C)** Interaction network for proteins that co-purified with CDC73_3YF adapted from an output from GeneMANIA using the proteins marked by solid borders as a search node. Solid lines connecting proteins indicate a reported physical interaction between two proteins. Circle sizes are proportional to the degree of connectivity to the proteins in the search node. **(D)** Total peptide spectrum matches (PSMs) for the PAF1c or proteins associated with H3K9 methyltransferase components generated from the AP-MS experiment for either CDC73 (sum of 2 replicates), CDC73_3YF (sum of 3 replicates), or MigR1 control (sum of 3 replicates).

To determine whether the proliferation deficient phenotype associated with CDC73_3YF was due to differences in protein-protein interactions, we performed Affinity Purification-Mass Spectrometry (AP-MS) to find the interactome of CDC73 and CDC73_3YF (Figure 2A). First, HEK293T cells were transiently transfected with FLAG tagged *CDC73_3YF* or wildtype *CDC73* and subjected to FLAG IP. Bait and co-purifying proteins were observed by Coomassie Blue staining, and differential banding patterns suggested different interactomes for CDC73 and CDC73_3YF (Figure 2B). To identify CDC73 interactions that are specifically relevant to AML, M1 murine AML cells were stably transduced with *FLAG-CDC73* or *FLAG-CDC73_3YF*. Co-IPs from M1 cells were analyzed using liquid chromatography tandem mass spectrometry (LC-MS/MS). Using the Contaminant Repository for Affinity Purification online resource (CRAPome), protein-protein interactions were scored using Significance Analysis of Interactome (SAINT) probabilistic scoring (Figure 2A, Supplementary Table 1). Interestingly, there was a distinct subset of 87 potential CDC73_3YF interacting proteins that were not found in the interactome of CDC73 (Supplementary Table 1). Analysis of this interactome using the GeneMANIA database to search for previously reported protein-protein interactions uncovered a group of interacting proteins in this group associated with transcriptional repression (Supplementary Figure 2A). We studied this group of proteins as a new search node in GeneMANIA and cross-referenced this protein interaction network with our AP-MS data. This identified a group of epigenetic modifying proteins that catalyze histone H3 lysine 9 methylation (H3K9me) that preferentially associated with CDC73_3YF (Figure 2C, 2D). These include: EHMT1 (GLP), EHMT2 (G9a) and SETDB1. In addition, several proteins associated with these H3K9 methyltransferases co-purified with CDC73_3YF, including WIZ, which associates with GLP and G9a; PML, which associates with GLP and SETDB1; and ATF7IP which associates with SETDB1 [43, 52, 53]. (Figure 2C, 2D).

**Figure 3:**
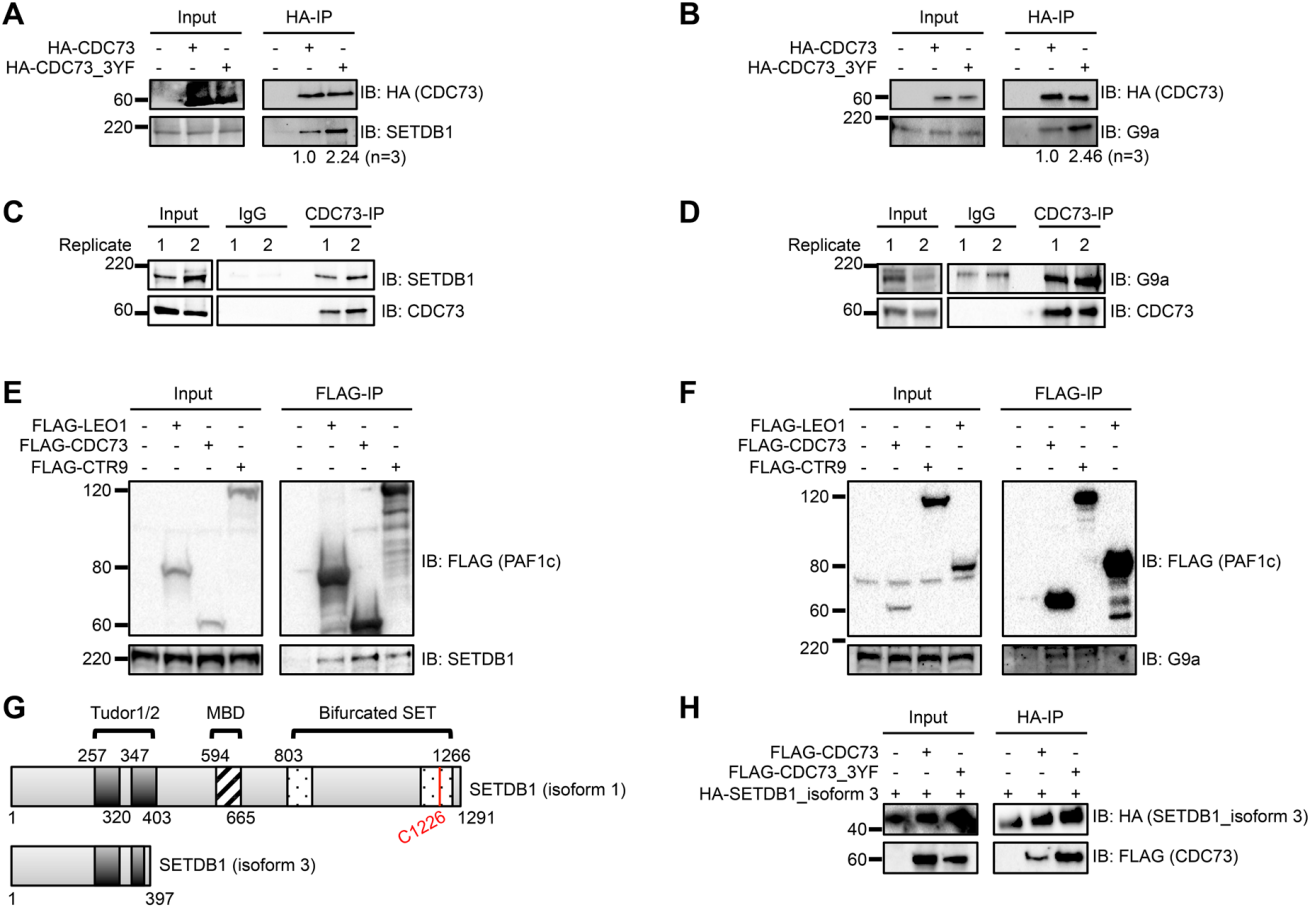
The PAF1c interacts with H3K9methyltransferases SETDB1 and G9a **(A-B)** HEK293T cells were transiently transfected with HA tagged CDC73 or CDC73_3YF. HA- IPs were performed and co-purifying H3K9 methyltransferase proteins were detected by western blotting with indicated antibodies. Densitometry was performed on H3K9methyltransferase bands normalized to the HA-IP bait bands. Shown is the average of n=3 quantifications. **(C-D)** CDC73-IPs were performed in THP-1 human AML cells and co-purifying H3K9 methyltransferase proteins were detected by western blotting with indicated antibodies. Two biological replicates are shown. **(E-F)** Flag tagged PAF1c components were transiently expressed in HEK293T cells. FLAG-IPs were performed and co-purifying proteins were detected with the indicated antibodies for H3K9 methyltransferases. Shown are representative blots of n=2-3 biological replicates. **(G)** Schematic showing the domains that are conserved between SETDB1 isoform 1 (top) and SETDB1 isoform 3 (bottom) as annotated in UniProt (Q15047) [69]. MBD = methyl CpG binding domain. **(H)** HA-tagged isoform 3 of SETDB1 and FLAG-CDC73 or FLAG-CDC73_3YF were transiently transfected in HEK293T cells. HA-IPs were performed and the CDC73 constructs were detected with a FLAG immunoblot. Shown is a representative blot of n=4 biological replicates. EV=Empty Vector control (MigR1).

CDC73 has previously been reported to interact with H3K9 methyltransferases G9a and SUV39H1. Yang *et al.* demonstrated that CDC73 recruits these methyltransferases to the *CCND1* promoter in HeLa cells and promotes H3K9 di- and tri- methylation (H3K9me2/3) [30]. For this study, we focused on H3K9 methyltransferases that were found specifically in our AP-MS data. Despite having the highest SAINT probability score, we were unable to validate an interaction between CDC73 and GLP, possibly related to antibody efficiency (data not shown). However, IP-western blots demonstrated that HA-CDC73 and HA-CDC73_3YF both pulled down endogenous SETDB1 and G9a in transiently transfected HEK293T cells. Consistent with the AP-MS data, there was a stabilized interaction between CDC73_3YF and SETDB1 or G9a compared to the interactions with CDC73 (Figure 3A, 3B). We confirmed this interaction between endogenous proteins in human AML THP-1 cells by subjecting cells to a CDC73-IP. Immunoblotting revealed an interaction between endogenous CDC73 and endogenous SETDB1 and G9a (Figure 3C, 3D). We were also interested in whether this interaction was a PAF1c dependent interaction or an independent function of CDC73. We therefore performed IPs on FLAG-tagged PAF1c components CTR9 and LEO1 in transiently transfected HEK293T cells. We found that CDC73, CTR9, and to a lesser degree LEO1 co-immunoprecipitate endogenous SETDB1 and G9a suggesting the interactions occur with the PAF1c (Figure 3E, 3F). Due to the novelty of the SETDB1 interaction, we focused our studies on SETDB1. To further confirm the stabilized interaction with CDC73_3YF and to determine the SETDB1 domains that are involved in the CDC73 interaction, we utilized a natural isoform of SETDB1 (isoform 3; NCBI reference sequence NP_001157114.1) that lacks the methyl binding domain (MBD) and the catalytic bifurcated SET domain (Figure 3G) [54, 55]. HEK293T cells were transiently transfected with *HA-hSETDB1*_isoform 3 and *FLAG-CDC73* or *FLAG-CDC73_3YF*. The cells were then subjected to HA-IPs and western blots. We observed that CDC73 copurified with the shorter isoform of SETDB1, and CDC73_3YF demonstrated a stronger co-purification (Figure 3H). Together, these data show that CDC73 interacts with at least SETDB1 and G9a and that CDC73_3YF stabilizes interaction with these H3K9 methyltransferases.

### SETDB1 modulates expression of PAF1c oncogenic target genes

**Figure 4:**
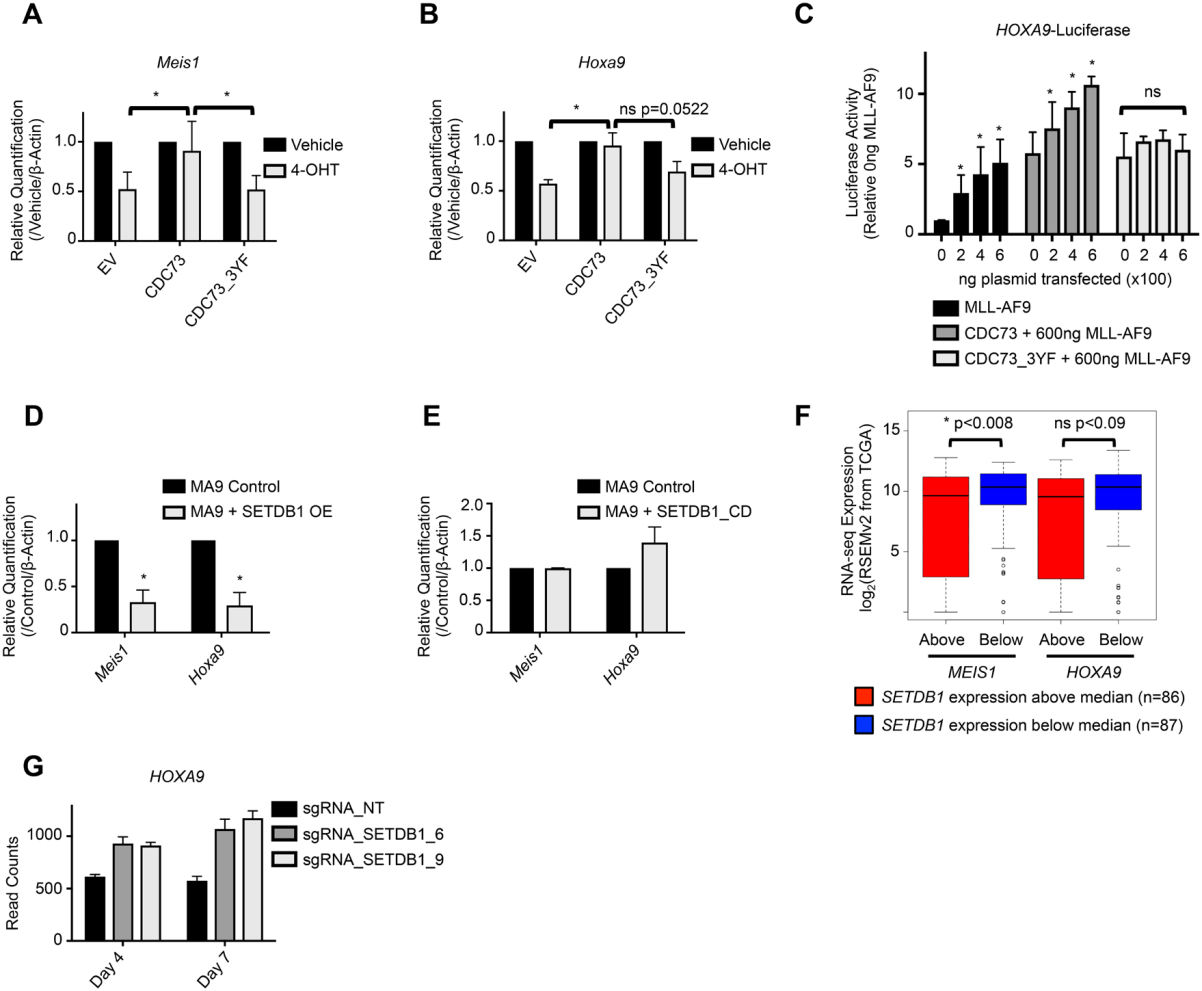
SETDB1 and CDC73_3YF mediated repression of *Hoxa9* and *Meis1* transcription **(A-B)** MLL-AF9 transformed *CDC73fl/fl*-CreER^T2^ CDC73 re-expression cells were plated in the presence or absence of 4-OHT and RNA was collected after 48 hours. qPCR was performed to detect expression of *Meis1* or *Hoxa9*. 4-OHT treated group was normalized to vehicle treated group for each cell type. Error bars represent standard deviations of the biological replicates (biological replicates n=5, n=3, respectively; 3 technical replicates each). Welch’s two-tailed unpaired t-test was used to compare the gene expression of the 4-OHT treated groups, using CDC73 re-expression as the control. **(C)** HEK293T cells were transiently transfected with the indicated plasmids, Hoxa9 luciferase, and Firefly-Renilla. Luminescence readings were taken at 48 hours and plotted relative to empty vector control transfections. Statistics were calculated using 2-way ANOVA with post-hoc Dunnett’s testing (biological replicates n=5; 3 technical replicates each). **(D-E)** qPCR detection of *Meis1* and *Hoxa9* expression in MLL-AF9 control cells or MLL-AF9 co-transduced with (D) SETDB1 or (E) SETDB1_CD overexpression vector. Welch’s two-tailed unpaired t-test was used to compare the gene expression relative to β-actin housekeeping gene of the SETDB1 overexpression cells compared to control. SETDB1 or SETDB1_CD overexpression groups were normalized to their concurrently made control cell line groups. Error bars represent standard deviations of the biological replicates (biological replicates n=2-3; 3 technical replicates each). **(F)** RNA-seq data, downloaded through the cBioPortal analytical tool, was deposited to the TCGA by Ley *et al.* [57, 70, 71]. Data was mined for expression correlation between *SETDB1* and *MEIS1* or *HOXA9*. Patient samples were divided into *SETDB1* expression higher than the median and those that were lower, and the gene of interest expression was plotted on the y-axis. Statistical significance was analyzed using the Mann-Whitney-Wilcoxon test. **(G)** RNA-seq data, downloaded through the Gene Expression Omnibus (GEO), was deposited by Cuellar *et al* [58, 72]. Data was mined for HOXA9 expression in human THP1 cells expressing Cas9 and one of two small guide RNAs (sgRNA_SETDB1_6 and sgRNA_SETDB1_9) designed to interfere with SETDB1 expression. Control cells expressed Cas9 and a non-targeting sgRNA (sgRNA_NT). RNA was collected and sequenced at two different time points- 4 days after introduction of the sgRNA and 7 days after introduction of the sgRNA. Each bar is the average of three biological replicates and the error bar represents the standard deviation. ^*^p<0.05; EV = Empty Vector control (MigR1); OE = Overexpression.

We hypothesized that the function of the PAF1c may be regulated, in part, via its interactions with SETDB1. To test this, we investigated transcription of PAF1c targets in AML cells expressing CDC73_3YF. We collected RNA from MLL-AF9-*Cdc73fl/fl*-CreER^T2^ cells expressing either CDC73 or CDC73_3YF treated with 4-OHT or vehicle. Interestingly, while CDC73 completely rescued expression of known PAF1c targets *Meis1* and *Hoxa9* upon 4-OHT induced deletion of *Cdc73*, CDC73_3YF was incapable of rescuing expression (Figure 4A, 4B). To more directly assess the effects of CDC73_3YF on transcription, we utilized a luciferase reporter construct. We used a *Hoxa9* luciferase reporter, which is a known MLL-AF9 target gene that is dependent on the PAF1c for full expression [10]. As expected, overexpressing CDC73 significantly augmented MLL-AF9 mediated transactivation of the *Hoxa9* promoter in a dose dependent manner (Figure 4C). Interestingly, overexpression of CDC73_3YF displayed no transcriptional synergy with MLL-AF9 in activating the *Hoxa9*-luciferase reporter (Figure 4C), consistent with *Hoxa9* transcript levels observed in CDC73_3YF expressing MLL-AF9 cells (Figure 4B). Due to the role of H3K9 methyltransferases in repressing gene transcription, we hypothesized that the transcriptional phenotype associated with *Cdc73*-/- cells re-expressing CDC73_3YF was due, in part, to H3K9 methyltransferase mediated transcriptional repression of known PAF1c target genes. Thus, we generated MLL-AF9 AML cell lines that overexpress human *MSCV-HA-SETDB1* (referred to hereafter as *SETDB1*) and MLL-AF9-*MSCV* control cell lines by retroviral transduction and collected RNA for gene expression analysis. qPCR experiments demonstrate reduced expression of *Meis1* and *Hoxa9* in MLL-AF9 cells overexpressing SETDB1 compared to control MLL-AF9 cells (Figure 4D). To determine whether the methyltransferase activity of SETDB1 is necessary for the reduction of *Meis1* and *Hoxa9* expression, we generated MLL-AF9 AML cell lines expressing the catalytic dead SETDB1_C1226A (SETDB1_CD) (Figure 3G) [56]. qPCR demonstrates that overexpression of this mutant did not lead to a significant change in *Meis1* or *Hoxa9* expression relative to MLL-AF9 control cells, suggesting an important role for the methyltransferase activity of SETDB1 in regulating the transcription of these genes (Figure 4E). To determine if increased expression of *SETDB1* correlates with the reduced expression of *MEIS1* and *HOXA9* in human AML samples, we mined RNA-seq data from 173 AML patients deposited in The Cancer Genome Atlas (TCGA) [57]. Consistent with our data, patient samples with *SETDB1* expression that was greater than the median expression of all samples in the set (n=86) had a significantly lower expression of *MEIS1* (Figure 4F). This trend held true for *HOXA9* expression, though the difference was not significant (Figure 4F). To explore the effect of SETDB1 loss of function on AML cells, we mined data from a recent study that utilized CRISPR-Cas9 targeting of SETDB1 in the human MLL-AF9 driven AML cell line, THP-1 [58]. Loss of SETDB1 protein in THP-1 cells leads to increased *HOXA9* expression at both four days and seven days after introduction of small guide RNAs (sgRNAs) targeting Cas9 to *SETDB1*, consistent with a repressive role for SETDB1 at the *HOXA9* locus (Figure 4G). These data suggest that SETDB1 overexpression leads to the reduced transcription of at least a subgroup of PAF1c target genes.

### Stabilization of the SETDB1-PAF1c interaction increases promoter H3K9me3

**Figure 5:**
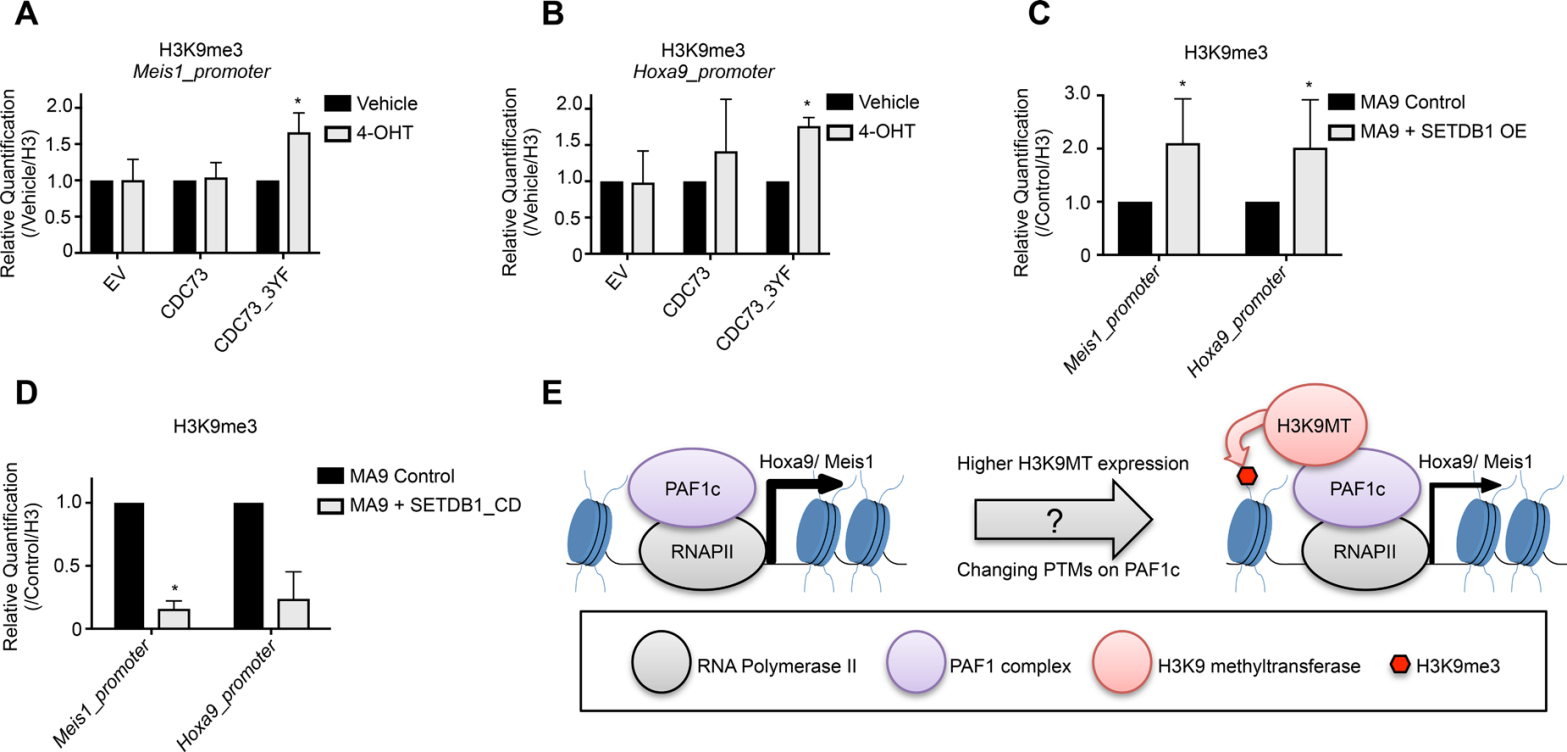
SETDB1 or CDC73_3YF contribute to epigenetic remodeling at the *Meis1* and *Hoxa9* promoter **(A-B)** ChIP-qPCR experiments for H3K9me3 at the (A) *Meis1* and (B) *Hoxa9* promoter were performed in MLL-AF9 transformed *CDC73fl/fl*-CreER^T2^ CDC73 re-expression cells treated with 4-OHT or vehicle control (biological replicates n=3; 2-3 technical replicates each). **(C-D)** ChIP-qPCR experiments for H3K9me3 at the *Meis1* and *Hoxa9* promoters were performed in MLL-AF9 control cells with and without transduced overexpressed (C) SETDB1 or (D) SETDB1_CD (biological replicates n=2-3; 2-3 technical replicates each). Statistical analysis was performed as described in the methods using a linear regression model with ANOVA found in Supplementary Figure 3. **(E)** Working model for the modulation of *Hoxa9* and *Meis1* transcription by the PAF1c-SETDB1 interaction. Increased expression of H3K9 methyltransferases or stabilized interaction between H3K9 methyltransferases and the PAF1c, possibly mediated by post-translational modifications (PTMs), leads to promoter H3K9 methylation and reduced expression of *Hox* genes. ^*^p<0.05; EV = Empty Vector control (MigR1); OE = Overexpression.

We next asked how the SETDB1-PAF1c interaction alters the epigenetic regulation of PAF1c target genes. We asked whether CDC73_3YF or overexpression of SETDB1 affected H3K9 methylation at the *Meis1* and *Hoxa9* gene promoters. To explore this we employed Chromatin Immunoprecipitation (ChIP) assays followed by qPCR on MLL-AF9 *Cdc73*-/- cells re-expressing CDC73 or CDC73_3YF. Cells expressing a control empty vector or re-expressing CDC73 did not display a difference in H3K9me3 at the *Meis1* promoter. However, cells re-expressing CDC73_3YF exhibited a significant increase in H3K9me3 at this locus (Figure 5A). Similarly, MLL-AF9 cells overexpressing SETDB1 showed an increase in H3K9me3 at the *Meis1* promoter compared to MLL-AF9 control cells (Figure 5C, Supplementary Figure 3A). We also performed ChIP-qPCR at the *Hoxa9* promoter. Here, loss of *Cdc73* in cells expressing the control vector exhibited little change in H3K9me3, whereas expression of CDC73_3YF again resulted in increased H3K9me3 (Figure 5B). We also observed that AML cells overexpressing SETDB1 had increased H3K9me3 at the *Hoxa9* promoter (Figure 5C, Supplementary Figure 3B). To determine whether the increase in H3K9me3 requires SETDB1 catalytic activity, we performed CHIP-qPCR in cells overexpressing SETDB1_CD. Surprisingly, these cells exhibited a significant loss of H3K9me3 at the *Meis1* and *Hoxa9* promoter regions (Figure 5D). This suggests that SETDB1 is responsible for the increase in H3K9me3 at these promoters in cells overexpressing SETDB1 and that SETDB1 may be responsible for depositing a basal level of H3K9me3 at the *Meis1* and *Hoxa9* promoter genes in resting state MLL-AF9 AML cells. We also tested for global changes in H3K9me3 using acid extracted histones. Western blots demonstrate that AML cells overexpressing SETDB1 have a marked increase in global H3K9me3 (Supplementary Figure 2B), however, neither loss of *Cdc73* nor re-expression of CDC73_3YF affected the global levels of H3K9me3 (Supplementary Figure 2C). These data may point to a more locus specific phenotype in CDC73_3YF cells compared to SETDB1 overexpressing AML cells. Together, this suggests the PAF1c may be regulated by interactions with SETDB1 that modulate H3K9me3 at target genes critical for leukemogenesis (Figure 5E).

## DISCUSSION

Here we describe a proteomics approach to identify interactions with the PAF1c subunit CDC73 that may play a critical role in the regulation of PAF1c functions. We found that a CDC73_3YF mutant, which displays enhanced interaction with β-catenin, surprisingly does not support AML cell growth or colony formation capacity, in contrast to its function in gastric carcinoma [47]. Using proteomic and bioinformatics analyses, we found that CDC73 interacts with G9a and SETDB1; an interaction that is stabilized by the CDC73_3YF mutant. These interactions with H3K9 methyltransferases elucidate a mechanism of PAF1c functional regulation that may explain recent reports of transcriptional repression associated with subunits of the PAF1c [26, 27]. Indeed, we demonstrate that interaction with SETDB1 modifies PAF1c mediated epigenetic and transcriptional regulation of leukemic target genes *Hoxa9* and *Meis1* in a manner dependent on SETDB1 catalytic activity (Figure 5E).

To determine the role of SETDB1 and the effects of enhancing the interaction between CDC73 and SETDB1 on modulating transcription of PAF1c target genes, we utilized two systems. First, we genetically deleted *Cdc73* and re-expressed wild type CDC73 or CDC73_3YF in MLL-AF9 AML cells. In the second, we stably overexpressed SETDB1 in mouse MLL-AF9 AML cells. We note that both CDC73_3YF re-expression and overexpression of SETDB1 leads to the reduced transcription of *Hoxa9* and *Meis1*, while the catalytically inactive SETDB1_CD does not affect transcription. This data is remarkably consistent with RNA-seq data mined from the TCGA, which shows that higher expression of *SETDB1* correlates to significantly lower expression of *MEIS1* and a trend downward in the expression of *HOXA9* [57]. Importantly, we also observe that promoter H3K9me3 is increased at the *Meis1* and *Hoxa9* loci in cells re-expressing CDC73_3YF or overexpressing SETDB1, while the SETDB1_CD overexpression leads to a reduction in H3K9me3 (Figure 5D). These data illustrate a novel interaction with SETDB1 that functionally modulates PAF1c activity through the SETDB1-mediated deposition of H3K9me3 and transcriptional repression.

Previous reports have demonstrated that phosphorylation of CDC73 at key tyrosine residues can act as a molecular switch to regulate interactions with β-catenin. In solid tissue tumors de-phosphorylation (mimicked using CDC73_3YF) enhanced the oncogenic activation of WNT targets by CDC73 [45, 47]. This prompted us to explore phosphorylation of CDC73 in AML cells. Interestingly, we were unable to observe phosphorylation on CDC73 in AML cells by IP western blots or mass spectrometry searches including phosphorylated peptides (data not shown). Therefore, it appears that phosphorylation of CDC73 may be dependent on cellular context. This raises interesting questions regarding the stabilization of the SETDB1 and G9a interaction with CDC73_3YF. Further studies will be necessary to determine what, if any, post-translational modifications are affected by mutating these three tyrosine residues in AML cells and, mechanistically, how this affects interactions with G9a and SETDB1 (Figure 5E).

CDC73 has previously been reported to interact with SUV39H1 and G9a proteins, though the authors were unable to validate the interaction between endogenous G9a and CDC73 [30]. Here we confirmed the interaction with G9a and uncovered a novel interaction with SETDB1 while also confirming a transcriptional consequence of these interactions at leukemogenic target loci. Additionally, we found that the interactions with SETDB1 and G9a occur in the context of the PAF1c and are not specific to the CDC73 subunit. Our AP-MS data also provides evidence that CDC73 may interact with GLP, though further studies are needed to validate this interaction. Taken together, these data provide varying degrees of evidence that the PAF1c binds to four different H3K9 methyltransferases. Thus, it is possible that the PAF1c binds to a previously reported multimeric H3K9 methyltransferase complex consisting of G9a, GLP, Suv39H1 and SETDB1 [39]. Further investigation is necessary to determine whether the PAF1c interacts with this H3K9 methyltransferase complex or individual H3K9 methyltransferases at specific loci. Given that CDC73 differs in its post translational modifications in a cell context manner, it will also be necessary to evaluate the nature of the PAF1c interaction with H3K9 methyltransferases in different cell types (Figure 5D). We must further investigate how this interaction affects different cellular phenotypes such as cell cycle and differentiation. Indeed, global increases in heterochromatin formation and H3K9 methylation are observed following differentiation of both embryonic stem cells and hematopoietic stem and progenitor cells [59–62]. The current study examined the role of SETBD1 in modulating the transcription of PAF1c target genes *Hoxa9* and *Meis1*. Due to the important role of Hoxa9 and Meis1 in development, normal hematopoiesis, and hematologic malignancies like AML, the interaction between SETDB1 and the PAF1c has major implications on our understanding of the regulation of these genes and the potential to therapeutically target these epigenetically regulated pathways.

### Experimental procedures

### Plasmid cloning and mutagenesis

Human *HA-SETDB1* isoform 1 was cloned into MSCVpuro retroviral vector and confirmed by sequencing. Human *FLAG-CDC73* and *HA-CDC73* were cloned into MigR1 retroviral vector and confirmed by sequencing. *MigR1-HA-CDC73_3YF, MigR1-FLAG-CDC73_3YF*, and *MSCVpuro-HA-SETDB1_C1226A* were generated using site-directed mutagenesis using the QuikChangeXL kit according to the manufacturer’s protocol (Agilent). *CMV-MYC-FLAG-PAF1, CMV-MYC-FLAG-CTR9*, and *CMV-MYC-FLAG-LEO1* were purchases from Origene.

### Cell line generation and cell culture conditions

Cell lines were generated from either wild type C57Bl/6 (Taconic Farms) mouse bone marrow, *Cdc73fl/fl*-CreER^T2^[51] mouse bone marrow, or M1 murine AML cell line as previously described [33]. Briefly, Platinum-E (Plat-E) viral packaging cells were transfected with *MSCVneo-MLL-AF9, MigR1-CDC73, MigR1-CDC73_3YF*, empty vector MigR1, *MSCVpuro-HA-SETDB1*, *MSCVpuro-HA-SETDB1_CD*, or empty vector MSCVpuro. Viral supernatants were collected and bone marrow or AML cells were spun with the virus and 5 ug/mL polybrene (Millipore). Cells transduced with MLL-AF9 were selected with 1 mg/mL neomycin. Cells transduced with *MigR1, CDC73*, or *CDC73_3YF* were sorted for GFP positivity. Cells transduced with *MSCVpuro, SETDB1*, or *SETDB1_CD* were selected with 1-2 ug/mL puromycin. All bone marrow-derived MLL-AF9 cells were cultured in IMDM supplemented with 15% Stem Cell FBS (Millipore), 1%pen/strep, and 10 ng/mL IL-3 (R&D). M1 murine AML cells were cultured in RPMI supplemented with 10% FBS and 1% pen/strep. HEK293T cells are grown in DMEM supplemented with 10% FBS.

### Proliferation assays and luciferase assays

MLL-AF9-*Cdc73fl/fl*-CreER^T2^ cells expressing *MigR1, MigR1-HA-CDC73*, or *MigR1-HA-CDC73_3YF* were seeded at 5x10^4^ cells in 2mL of normal growth media containing either 2.5 nM 4-hydroxytamoxifen (4-OHT) or an equivalent percentage ethanol vehicle control (vehicle). Viable cell number was counted each day for 4 days using Trypan Blue. On day 2, the cells were supplemented with fresh media, IL-3, and 4-OHT or vehicle. Luciferase assays were performed using the Dual Luciferase assay kit and a GloMax 20/20 Luminometer (Promega) as previously described [10].

### Colony formation assays

MLL-AF9-*Cdc73fl/fl*-CreER^T2^ cells expressing *MigR1, MigR1-HA-CDC73*, or *MigR1-HA-CDC73_3YF* grown in normal growth media were pretreated with 2.5 nM 4-OHT or an equivalent percentage vehicle for 24 hours. They were then seeded at a density of 1x10^3^ cells in 2mL semi-solid methylcellulose medium for mouse cells (STEMCELL M3234) containing 10 ng/mL IL-3. Colonies were counted and 1x images of the 5-phenyl tetrazolium chloride (INT) stained dishes were taken after 7 days of growth.

### Immunoprecipitations (IP) and antibodies

For HEK293T transient transfection experiments, cells are transfected following the Fugene^®^6 (Promega). Cells were collected, lysed and IP’d with anti-HA high affinity beads (Roche) or M2 anti-FLAG magnetic beads (Sigma). Antibodies used for western blotting were anti-HA (Abcam9110), anti-FLAG (Sigma F7425), anti-SETDB1 (Abcam107225, Bethyl A300-121), from Bethyl: anti-EHMT2 (A301-642), anti-PAF1 (A300-172), anti-CTR9 (A301-395), anti-LEO1 (A300-174), or anti-WDR61 (A305-191). Antibodies used for western blotting and ChIP were anti-H3 (Abcam1791, western blot 1:4000), anti-H3K9me3(Abcam 8898), anti-rabbitIgG (Millipore 12-370). All antibodies were generated in rabbit and were used at a dilution of 1:1000 for western blot and 4 ug/reaction for ChIP unless otherwise noted.

### Affinity purification-mass spectrometry

1x10^9^ cells M1 murine AML cells that stably express FLAG-CDC73 (n=2) or FLAG-CDC73_3YF (n=3) were harvested and lysed in 300mM KCl lysis buffer containing protease inhibitors and IGEPAL CA-630. Lysates were incubated with M2 FLAG magnetic beads (Sigma). Beads were washed 6 times with 0.3M-1M KCl and eluted with 15 ug 3X FLAG peptide. Proteins were denatured in 8M urea. Cysteines were reduced with 10 mM DTT and alkylated using 50 mM chloroacetamide. Proteins were digested with 500 ng of sequencing grade, modified trypsin (Promega). Reaction was terminated by acidification with trifluoroacetic acid (0.1% v/v) and peptides were purified using SepPak C18 cartridge following manufacturer’s protocol (Waters Corp) and dried. Peptides were reconstituted in HPLC loading buffer and resolved on a nano-capillary reverse phase column (Acclaim PepMap C18, 2 micron, 50 cm, ThermoScientific) using 0.1% formic acid/acetonitrile gradient at 300 nl/min (2-25% acetonitrile in 105 min; 25-40% acetonitrile in 20 min followed by a 90% acetonitrile wash for 10 min and a further 30 min re-equilibration with 2% acetonitrile) and directly introduced in to *Orbitrap Fusion Tribrid* mass spectrometer (Thermo Scientific, San Jose CA). MS1 scans were acquired at 120K resolution (AGC target=2e^5^, max IT=50ms). Data-dependent high-energy C-trap dissociation MS/MS spectra were acquired for the most abundant ions for 3 seconds following each MS1 scan (15K resolution; AGC target=5e^4^; relative CE ∼32%). Proteins were identified by searching the data against *Mus musculus* (Swissprot, v2016-04-13) using SEQUEST-HT (Proteome Discoverer v2.1, Thermo Scientific). Search parameters included MS1 mass tolerance of 10 ppm and fragment tolerance of 0.05 Da; two missed cleavages were allowed; carbamidimethylation of cysteine was considered fixed modification and oxidation of methionine, deamidation of asparagine and glutamine, phosphorylation of serine, threonine and tyrosine were considered as potential modifications. False discovery rate (FDR) was determined using Percolator and proteins/peptides with an FDR of ≤1% were retained for further analysis. The mass spectrometry proteomics data have been deposited to the ProteomeXchange Consortium via the PRIDE partner repository with the dataset identifier PXD009439 [73].

### Scoring of protein-protein interactions

Interactions with CDC73 and CDC73_3YF were scored using MS2 spectral counting (PSM counts). Using PSM counts as measures of protein abundance in each sample, SAINT probabilities for each interaction were calculated using the CRAPome online resource [63–65]. 3 FLAG-IP replicates of cells expressing only the empty vector MigR1 were used as controls. SAINT calculates the probability that an interaction is a true positive using a model where true-positive and false-positive interactions for each bait have distinct Poisson distributions. A value of 1 indicates a high probability of a bona-fide interaction [63]. SAINT parameters used were: average=best 2 replicates; virtual controls=10; iter(2000,4000); normalization=1. SAINT probabilities for all identified proteins are found in Supplementary Table 1. Proteins with a SAINT probability of >=0.7 for either or both bait proteins were kept as potential interacting proteins. We began our filtering with this relatively low threshold due to the low IP efficiency of CDC73_3YF relative to CDC73 and the possibility that our phenotypic effects were due to a more transient interaction. For the discovery of protein interactions involved in transcriptional repression that were potentially bound specifically to CDC73_3YF, all prey proteins with a SAINT probability >=0.7 were analyzed using GeneMANIA [66]. The network of interactions derived using GeneMANIA was filtered to include only those proteins with a published physical protein-protein interaction, including those contained in the BioGRID interaction database [67]. This protein-protein interaction network was then investigated to observe biologically interesting interaction subnetworks. When an interesting sub-network contained at least three proteins with SAINT probability > 0.7, that sub-network was used as a new search node for GeneMANIA. Protein-protein interaction networks shown in Supplementary Figure 2 were generated with GeneMANIA. Networks shown in Figure 1 were adapted from GeneMANIA using Microsoft PowerPoint 2011.

### Quantitative PCR (qPCR) for gene expression

RNA was harvested from MLL-AF9 + MSCVpuro control cells, MLL-AF9 + SETDB1, or MLL-AF9 + SETDB1_CD overexpression cells; or from MLL-AF9-Cdc73fl/fl-CreER^T2^ MigR1, CDC73, or CDC73_3YF cells treated with 2.5-5 nM 4-OHT or vehicle control. 1-5x10^6^ cells were harvested and RNA was extracted using the Qiagen RNeasy mini plus kit. cDNA synthesis was performed using oligo-dT priming and the SuperScript III kit (Invitrogen). qPCR was performed using the fast SYBR-green mastermix protocol (Thermo Fisher). Primer sets used were: *Meis1*_F-5’ATCAGAGCGCCAGGACCTAT3’; *Meis1*_R- 5’CTTCCCCCTGGCTTTCGATT3’; *Hoxa9*_F- 5’GAATGAGAGCGGCGGAGAC3’; *Hoxa9*_R- 5’GAGCGAGCATGTAGCCAGTTG3’; *β-Actin*_F- 5’GCCCTGAGGCTCTTTTCCAG3’; *β-Actin_R*- 5’TGCCACAGGATTCCATACCC3’

### Chromatin immunoprecipitation-qPCR (ChIP-qPCR)

ChIP experiments were performed as previously described in MLL-AF9 + MSCVpuro control cells, MLL-AF9 + SETDB1, or MLL-AF9 + SETDB1_CD cells; or in MLL-AF9-Cdc73fl/fl-CreER^T2^ MigR1, CDC73, or CDC73_3YF cells treated with 2.5-5 nM 4-OHT or vehicle control [68]. Briefly, 3x10^7^ AML cells were crosslinked with 1% formaldehyde, lysed with 1% SDS and sonicated on a Bioruptor Pico sonication device (Diagenode). Cleared lysates were immunoprecipitated with anti-Histone H3 or anti-Histone H3 (trimethylated K9) using Protein G dynabeads. The IPs were washed with a low salt buffer, a high salt buffer, and a stringent lithium chloride wash buffer. Protein-DNA complexes were eluted in 1% SDS, were decrosslinked in high salt and treated with RNaseA and ProteinaseK. DNA was purified with a Qiagen PCR purification kit. qPCR was performed using the fast SYBR-green mastermix protocol. Primer sets used were: *Meis1*_promoter_F-5’TCAAAGTGACAAAATGCAAGCA3’; *Meis1*_promoter_R- 5’CCCCCCGCTGTCAGAAG3’; *Hoxa9*_promoter_F-5’TGACCCCTCAGCAAGACAAAC 3’; *Hoxa9*_promoter_R- 5’TCCCGCTCCCCAGACTG 3’

### Statistical analysis of ChIP-qPCR data

Due to the high variability in ChIP-qPCR ratios from “test” cells over “control” cells related to technical efficiency of ChIP experiments, ChIP-qPCR data were analyzed for statistical significance using ANOVA on fitted linear models. ANOVA models in SAS (PROC GLM) or R (lm) were used to compare treated and control groups. The model includes group (treated/control), cell lines and the interaction between group and cell lines. If the interaction is not significant, we report the p-value for the main effect of group. Significance is determined if p<0.05. All analyses were conducted using SAS (version 9.4, SAS Institute, Cary, NC) or R. All graphs were generated in R or Prism (version 7.0c). All technical replicate (3 per biological replicate) values were included for statistical analyses.

## SUPPLEMENTARY MATERIALS FIGURES AND TABLE




