# Supplementary material for: PAF1 complex interactions with SETDB1 mediate promoter H3K9 methylation and transcriptional repression of Hoxa9 and Meis1 in acute myeloid leukemia
Source: Oncotarget. 2018 Apr 24;9(31):22123–36. doi: 10.18632/oncotarget.25204 (PMC5955148; doi:10.18632/oncotarget.25204)
Supplement: Supplementary file 1 [file oncotarget-09-22123-s001.pdf]

PAF1 complex interactions with SETDB1 mediate promoter H3K9 methylation and transcriptional repression of *Hoxa9* and *Meis1* in acute myeloid leukemia

SUPPLEMENTARY MATERIALS

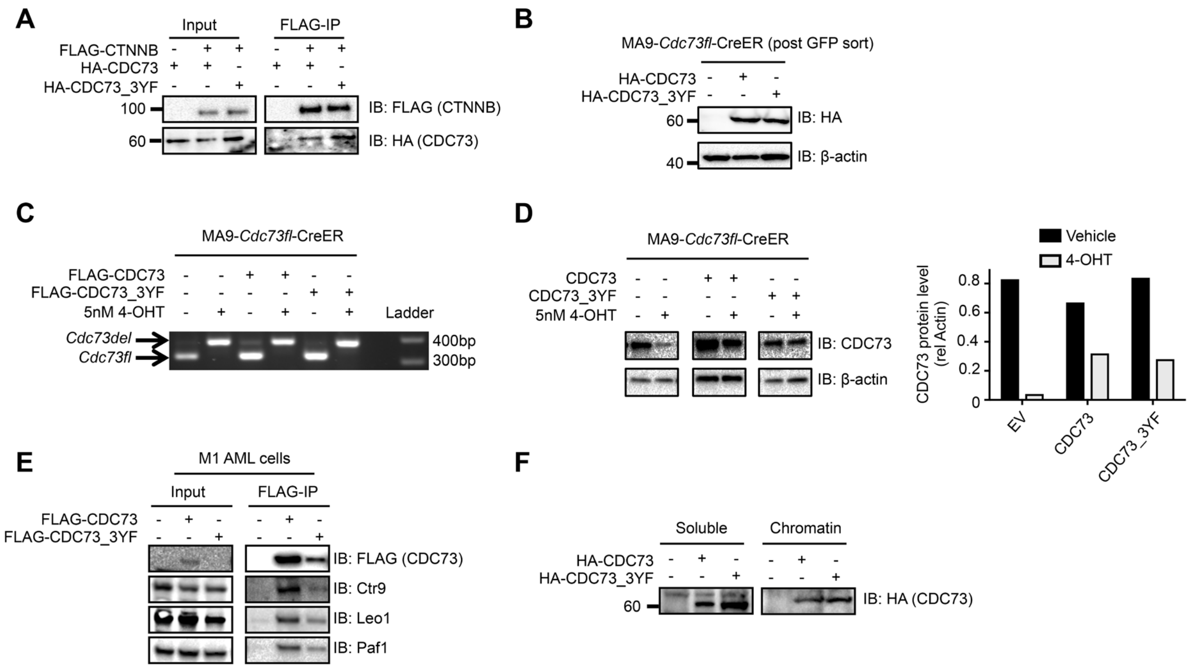

Supplementary Figure 1: (A) HEK293T cells were transiently co-transfected with either HA-CDC73 or HA-CDC73_3YF and FLAG-CTNNB. FLAG-IPs were performed and immunoblotted with the indicated antibodies. **(B)** Western blot of whole cell lysates from MLL-AF9 transformed *CDC73fl/fl*-CreER^{T2} CDC73 or CDC73_3YF re-expression cells and immunoblotted with the indicated antibodies demonstrating expression of HA-CDC73 and HA-CDC73_3YF. **(C)** Genotyping of MLL-AF9-*Cdc73fl/fl*-CreER^{T2} cells treated with 4-OHT or vehicle control to confirm genetic excision of the *Cdc73* allele. **(D)** Whole cell lysates from MLL-AF9-*Cdc73fl/fl*-CreER^{T2} cells treated with 4-OHT or vehicle control immunoblotted with anti-CDC73 antibody (left). Densitometry was performed on CDC73 protein bands and values were plotted relative to β-actin protein band signal (right). **(E)** M1 mouse AML cells that express stable retroviral FLAG-CDC73 or FLAG-CDC73_3YF are subjected to FLAG-IPs. PAF1c proteins are detected using western blotting with the indicated antibodies. **(F)** HEK293T were transiently transfected with CDC73 or CDC73_3YF. Whole cell lysates were collected and run as soluble fraction. Cells were lysed, the insoluble chromatin fraction was spun down and washed three times, then immunoblotted with HA antibody as the chromatin fraction.

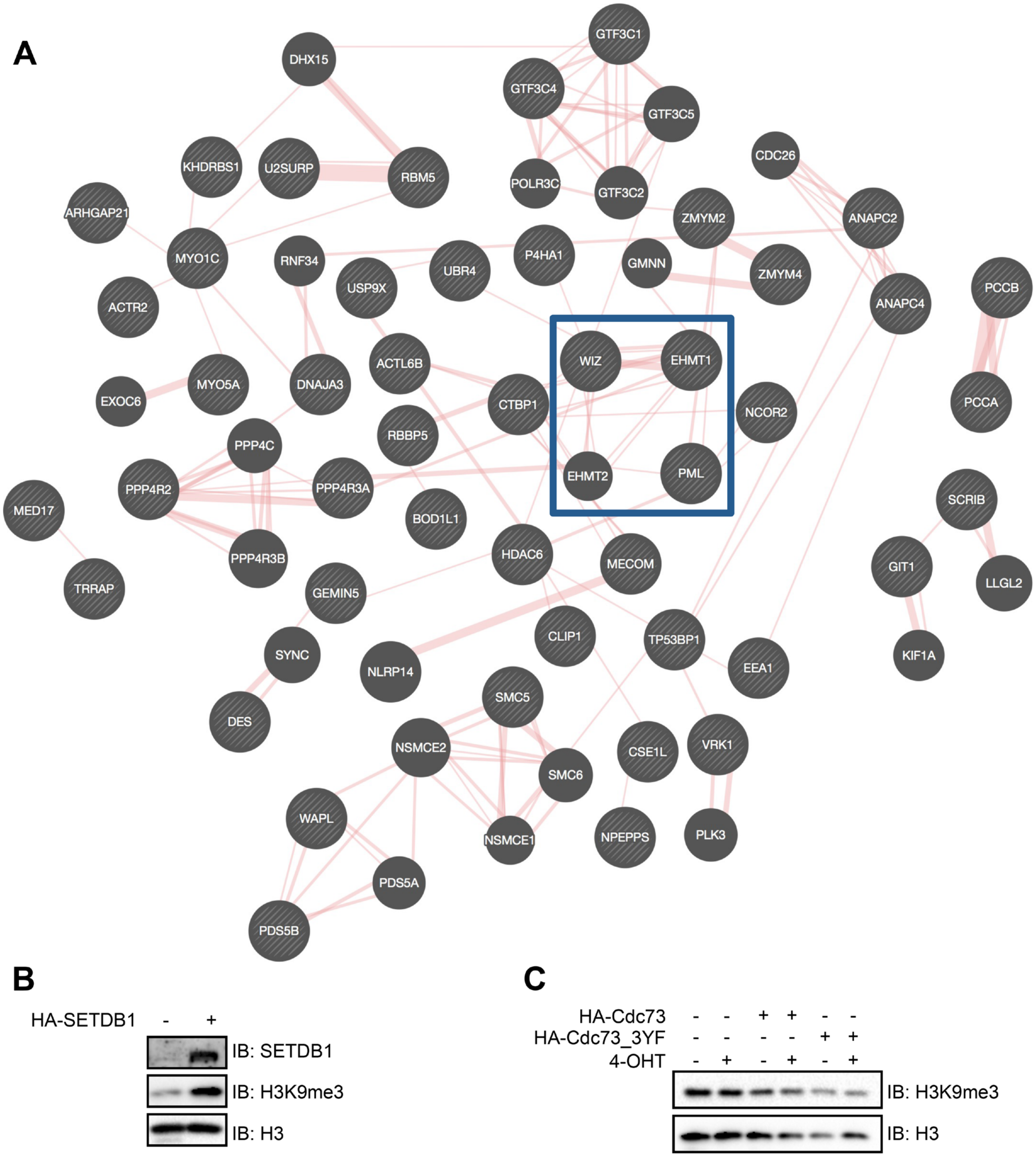

Supplementary Figure 2: (A) Interaction network output by GeneMANIA for proteins that co-purified with CDC73_3YF in the AP-MS experiment. The interactions were filtered to include only those described as physical interactions. Circles containing diagonal lines indicate proteins included in the search node. The blue rectangle indicates the sub-network of proteins that was found to be associated with transcriptional repression. The proteins contained in this rectangle were used in the subsequent targeted GeneMANIA search. **(B)** Western blot of acid extracted histones from MLL-AF9 control cells or MLL-AF9 cells co-transduced with SETDB1 overexpression vector. Western blots were blotted with the indicated antibody. The top panel is a whole cell lysate demonstrating overexpression of SETDB1. **(C)** Western blots of acid extracted histones from MLL-AF9 transformed *CDC73fl/fl*-CreER^{T2} CDC73 re-expression cells. Western blots were blotted with the indicated antibody.

A

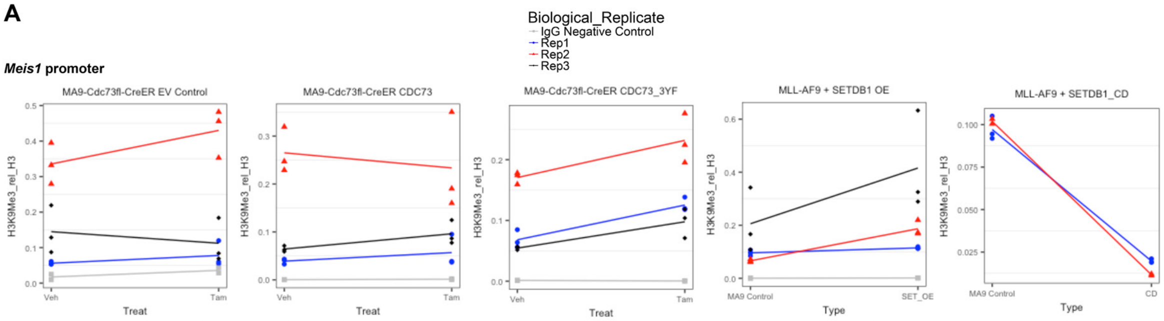

	Sum Sq	F value	Pr(>F)
Treat	0.00379	1.48	0.243
Rep	0.427	55.6	5.06e-08
Treat:Rep	0.0123	1.6	0.234

	Sum Sq	F value	Pr(>F)
Treat	0.000143	0.0688	0.797
Rep	0.192	30.8	2.04e-06
Treat:Rep	0.00335	0.538	0.664

	Sum Sq	F value	Pr(>F)
Treat	0.0106	27.3	0.000129
Rep	0.104	89.1	2.33e-09
Treat:Rep	0.00273	2.33	0.118

	Sum Sq	F value	Pr(>F)
Type	0.0503	6.84	0.0203
Rep	0.259	11.7	0.000408
Type:Rep	0.0384	1.74	0.205

	Sum Sq	F value	Pr(>F)
Type	0.017	980	7.05e-08
Rep	6.52e-06	0.375	0.563
Type:Rep	9.85e-05	5.67	0.0547

B

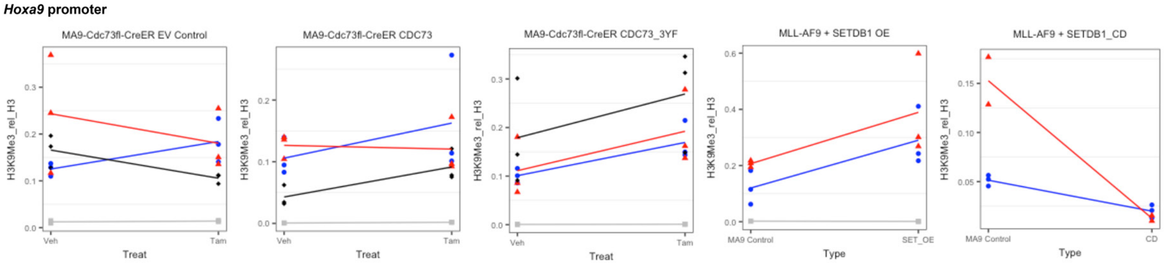

	Sum Sq	F value	Pr(>F)
Treat	0.00165	0.484	0.498
Rep	0.0966	9.47	0.00113
Treat:Rep	0.0149	1.47	0.266

	Sum Sq	F value	Pr(>F)
Treat	0.00411	2.15	0.165
Rep	0.0531	9.25	0.00126
Treat:Rep	0.00436	0.759	0.535

	Sum Sq	F value	Pr(>F)
Treat	0.0236	4.81	0.0456
Rep	0.121	8.23	0.00211
Treat:Rep	0.00553	0.377	0.771

	Sum Sq	F value	Pr(>F)
Type	0.0699	7.29	0.0223
Rep	0.213	11.1	0.00287
Type:Rep	0.0238	1.24	0.331

	Sum Sq	F value	Pr(>F)
Type	0.014	63.1	0.000212
Rep	0.0053	23.9	0.00275
Type:Rep	0.00705	31.8	0.00134

Supplementary Figure 3: Linear regression models were run to test the difference between treated groups or MLL-AF9 control vs MLL-AF9 + SETDB1 or MLL-AF9 + SETDB1_CD overexpression for ChIP-qPCR at the **(A)** *Meis1* or **(B)** *Hoxa9* promoter locus. Technical replicate values are represented as the same color, whereas biological replicates are indicated by different colors. ANOVA tables are shown below the interaction plots generated by the linear model. Type=Cell Line, Rep=Biological_Replicate, Treat:Rep=Interaction.

Supplementary Table 1: xlsx document containing all proteins inferred from AP-MS data as well as statistical analysis for confidence of potential interactions and the comparison of CDC73 and CDC73_3YF interactomes.
The bait/ prey proteins are annotated with a PROTID corresponding to the UniProt ID for the given bait/ prey protein, a Gene Symbol corresponding to the gene that codes for the identified protein, and a Gene Name that corresponds to the full name of that gene/ protein that was identified.

Sheets:

- **PSM_Scoring** contains all spectral counting values for all replicates of CDC73, CDC73_3YF, and Control AP-MS experiments.
- **All_SAINT_probabilities** contains all SAINT probabilities (SP) calculated for the AP-MS experiments using the CRAPome online tool.
- **Overlapping_proteins** contains a list of proteins that are found in the interactome of both CDC73 and CDC73_3YF with a SAINT probability of >=0.7.
- **CDC73_3YF_enriched** contains a list of proteins that are found in the interactome of CDC73_3YF with a SAINT probability of >=0.7 and have a SAINT probability of <= 0.7 in the CDC73 AP-MS.
- **CDC73_enriched** contains a list of proteins that are found in the interactome of CDC73 with a SAINT probability of >=0.7 and have a SAINT probability of <= 0.7 in the CDC73_3YF AP-MS.
- **Key** contains a brief definition for any abbreviations used in the other sheets.

See Supplementary File 1
